# Supplementary figures and images for: In situ Patch-seq analysis of microglia reveals a lack of stress genes as found in FACS-isolated microglia
Source: PLoS One. 2024 Jul 11;19(7):e0302376. doi: 10.1371/journal.pone.0302376 (PMC11239014; doi:10.1371/journal.pone.0302376)

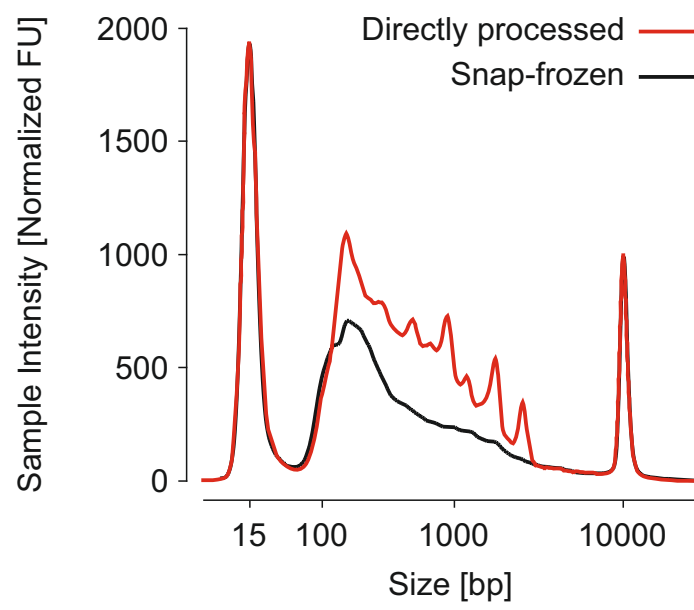

Suppl. Figure 1

Supplement: S1 Fig — Red profile was obtained from a sample that was processed directly after harvesting the cytosol whereas the black sample was snap-frozen in liquid N2 for 24 hours before the SmartSeq2 protocol was applied. (PDF) [file pone.0302376.s001.pdf]

**A**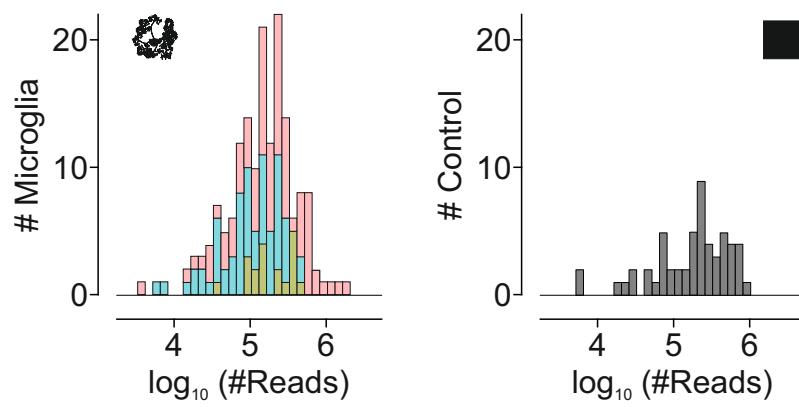**B**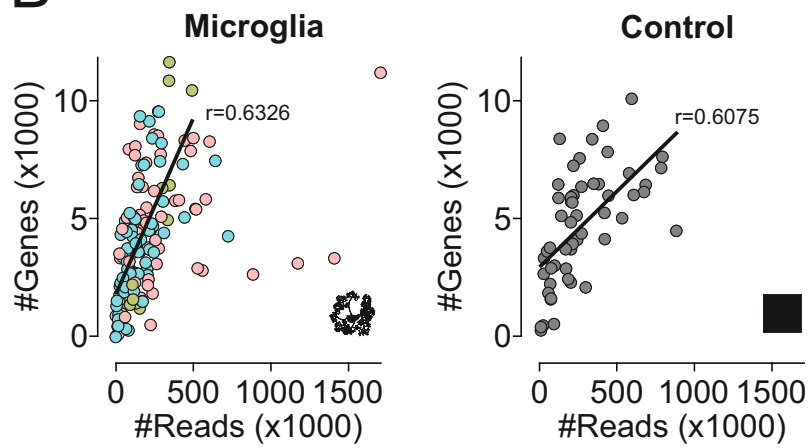**C**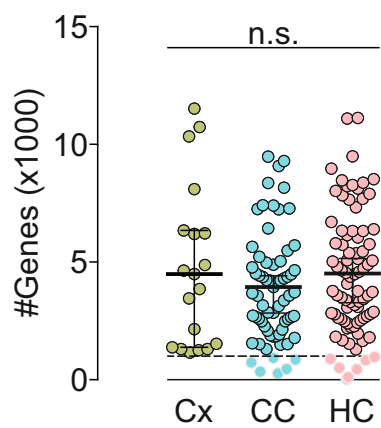

Supplement: S2 Fig — A Histograms depicting the number of sequenced reads obtained in MG (left) and EC (right) samples. B Correlation of read counts and gene numbers in MG (left) and EC (right) samples. C Number of identified genes per brain region. Note that samples with less than 1000 Genes were excluded from analysis. Significance was tested by Kruskal-Wallis followed by a Dunn´s test. P-values were as followed: Cx-CC:>0.9999, Cx-HC:>0.9999, CC-HC:0.3456. (PDF) [file pone.0302376.s002.pdf]

**A**

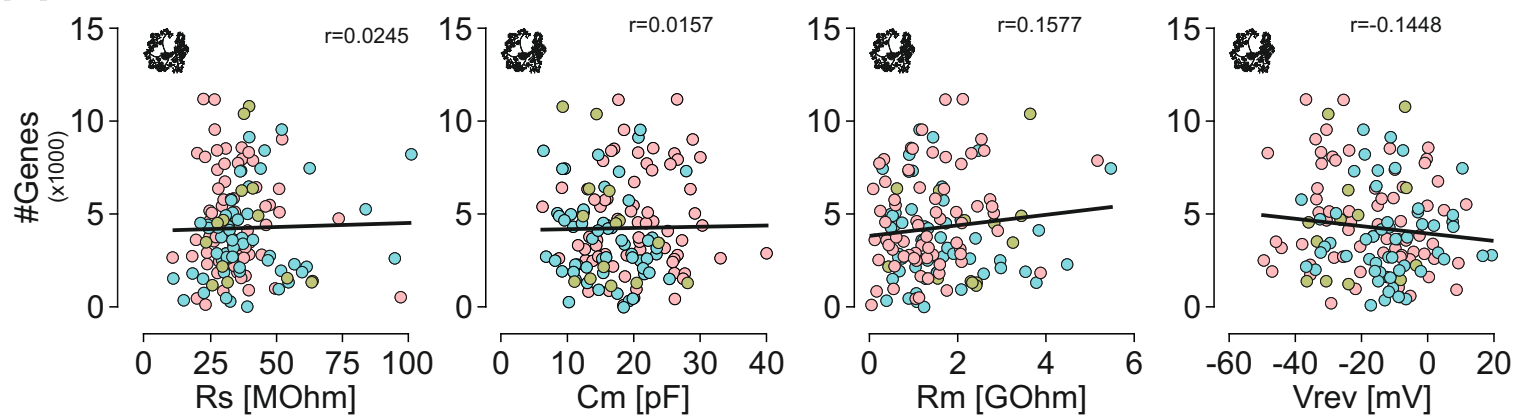

**B**

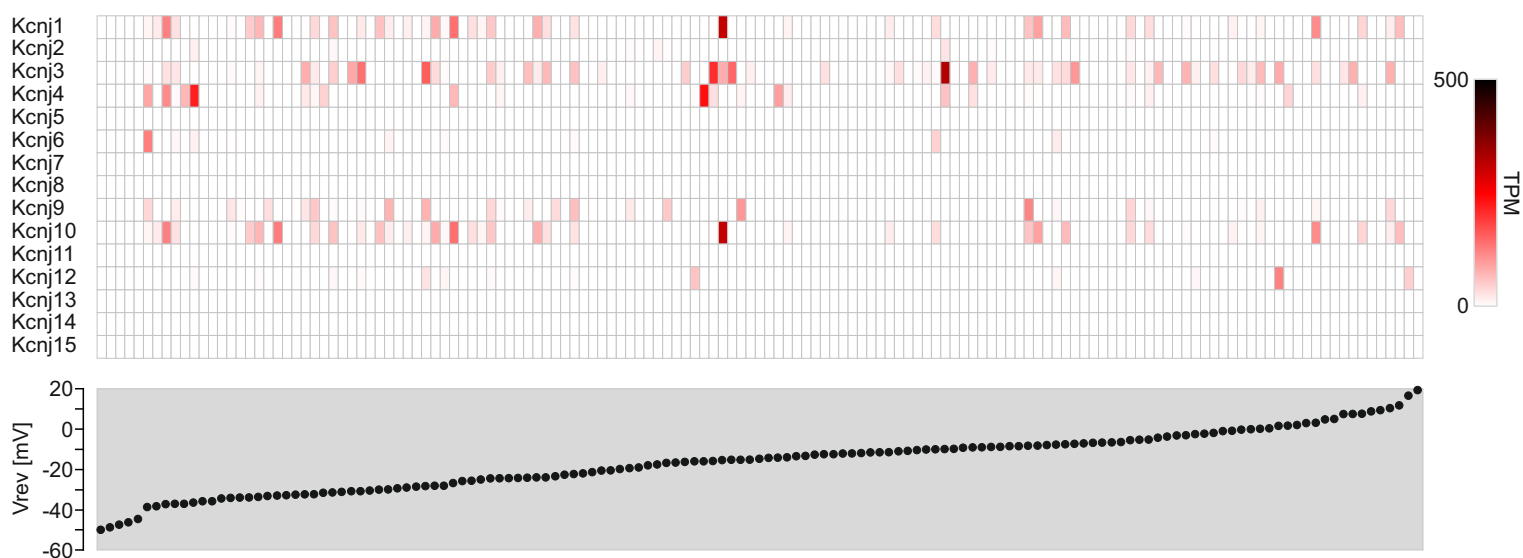

**C**

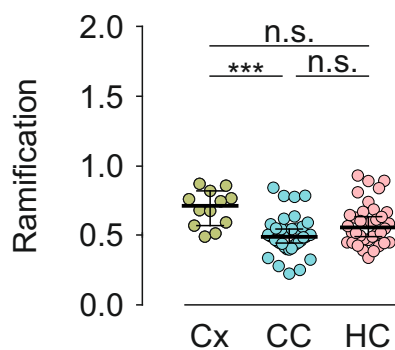

**D**

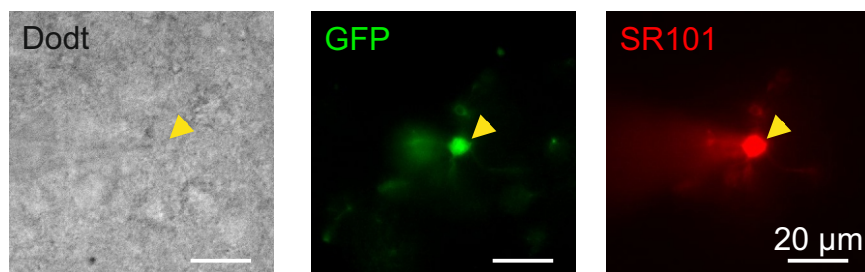

Supplement: S3 Fig — A Correlation between series resistances (Rs; left), capacities (Cm; middle left), membrane resistances (Rm; middle right) and reversal potentials (Vrev; right) with gene numbers in microglia from cortex, hippocampus and corpus callosum. None of the electrophysiological properties clearly correlated with the number of genes obtained in the subsequent single cell analysis. B Heatmap shows the expression of Kir channel family genes (Kcnj1-15) in patched-clamped microglia from cortex, hippocampus and corpus callosum. Samples are ordered due to the microglia reversal potentials where Vrev increases from left to right (see graph on the bottom). Note that there was no apparent correlation between Kir expression and Vrev. C Significance was tested by Kruskal-Wallis followed by a Dunn´s test. P-values were as followed: Cx-CC: 0.0004, Cx-HC:>0.0801, CC-HC:0.0780. D Sample in situ image of a microglia 5 min after breaking in. Intracellular solution contained SR101 in this experiment. Note the apparent ramified morphology of the patched microglia in GFP and SR101 channels. (PDF) [file pone.0302376.s003.pdf]

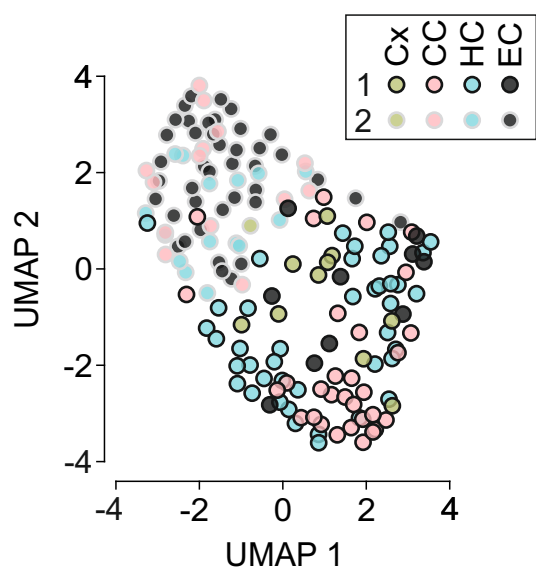

Suppl. Figure 4

Supplement: S4 Fig — UMAP plot of MG (yellow = cortex; red = corpus callosum; blue = hippocampus) and EC (gray) samples. Clustering analysis revealed two different clusters which are indicated by the black (Cluster 1) and gray (Claster2) outlines. (PDF) [file pone.0302376.s004.pdf]

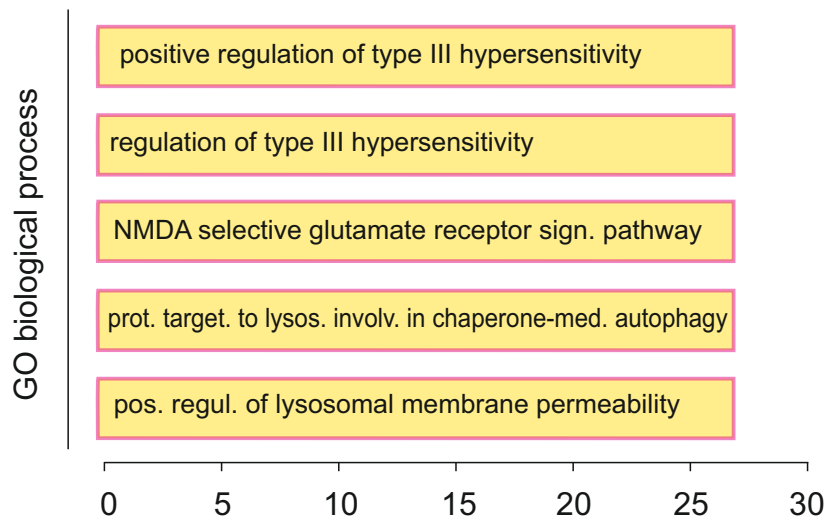

Suppl. Figure 5

Supplement: S5 Fig — Gene ontology (GO) enrichment analysis of marker-genes of Clusters 1 and 4 from Fig 4A. The bar chart represents the top 5 significantly enriched pathways. X-axis shows the fold enrichment of each pathway. (PDF) [file pone.0302376.s005.pdf]
